# Translation and Validation of the Chinese Version of the Rapid Geriatric Assessment (C-RGA): A Screening Tool for Geriatric Syndromes in Nursing Home Residents

**DOI:** 10.3390/nu17050873

**Published:** 2025-02-28

**Authors:** Jia Liu, Azera Hasra Ismail, Roszita Ibrahim, Yuezhi Zhu, Nor Haty Hassan

**Affiliations:** 1 Department of Nursing, Faculty of Medicine, Universiti Kebangsaan Malaysia, Kuala Lumpur 56000, Malaysia; 2 Department of Public Health Medicine, Faculty of Medicine, Universiti Kebangsaan Malaysia, Kuala Lumpur 56000, Malaysia; 3 Department of Biochemistry, Faculty of Medicine, Universiti Kebangsaan Malaysia, Kuala Lumpur 56000, Malaysia

**Keywords:** validity, reliability, RGA, nursing home, older residents

## Abstract

**Background**: Frailty, sarcopenia, nutritional risk, and cognitive impairment are prevalent geriatric syndromes that adversely affect health outcomes in older adults, underscoring the need for an effective screen tool to enable early detection and timely intervention. **Methods**: This study employed a cross-sectional validation design and translated, culturally adapted, and validated the Chinese version of the Rapid Geriatric Assessment (C-RGA) among 416 nursing home residents. The C-RGA consists of four subscales: the simple frail questionnaire screening tool (FRAIL), SARC-F for sarcopenia (SARC-F), the Simplified Nutritional Assessment Questionnaire (SNAQ), and the Rapid Cognitive Screen (RCS). **Results**: The C-RGA demonstrated high content validity (S-CVI/Ave = 0.982) and strong internal consistency (Cronbach’s α = 0.839). Factor analysis confirmed its four-domain structure, accounting for 61.497% of the variance. Model fit indices demonstrated good construct validity (χ^2^/*df* = 1.122, RMSEA = 0.024, GFI, AGFI, and CFI > 0.90), supporting the robustness of the assessment tool. Pearson correlation analysis revealed a strong association between FRAIL and SARC-F with SNAQ (r = −0.671, 95% CI: [−0.742, −0.600], *p* < 0.01) and a moderate correlation with RCS (r = −0.426, 95% CI: [−0.513, −0.339], *p* < 0.01), underscoring the interplay among nutritional deficits, muscle weakness, and cognitive impairment. **Conclusions**: The C-RGA demonstrates strong psychometric properties, supporting its potential use as a screening tool for the early detection of frailty, sarcopenia, nutritional risk, and cognitive impairment among nursing home residents, enabling timely and targeted interventions. Future research should further assess its applicability across diverse healthcare settings to enhance its generalizability and clinical utility.

## 1. Introduction

Population aging represents a significant public health challenge in China, with the population of adults aged 60 and above projected to surpass 402 million by 2040 [1]. Age-related syndromes, including frailty, sarcopenia, malnutrition, and cognitive impairment, substantially contribute to functional decline, an increased healthcare burden, and diminished quality of life. Early identification of these health risks is crucial for enabling timely interventions and optimizing health outcomes in older adults [2,3,4,5,6].

Various screening tools have been validated for geriatric assessment in China. Some screening tools, including Fried’s Frailty Phenotype [7], SARC-F for sarcopenia (SARC-F) [8], the Mini-Nutritional Assessment (MNA) [9], the Simplified Nutritional Appetite Questionnaire (SNAQ) [10], and the Short-Form Montreal Cognitive Assessment (MoCA) [11], primarily target single health domains, independently assessing frailty, sarcopenia, nutritional status, or cognitive function. Other tools, such as the Japan Frailty Scale (JFS-C) [12], are primarily designed for frailty screening, while also incorporating elements related to general health and functional status. However, most of these tools do not offer a comprehensive assessment of overall geriatric health status. The Comprehensive Geriatric Assessment (CGA) is widely regarded as the gold standard for assessing the overall health status of older adults [4]. However, its complexity and time-intensive nature restrict its feasibility for large-scale screening, particularly in institutional care and primary healthcare settings [5].

The Rapid Geriatric Assessment (RGA), developed by Morley’s team at Saint Louis University based on the CGA, was designed as an efficient and practical screening tool that concurrently evaluates frailty, sarcopenia, nutritional risk, and cognitive function, thereby addressing the limitations of existing tools. Compared to CGA, RGA maintains multidimensional assessment capabilities while significantly reducing assessment time, thereby improving its feasibility for large-scale screening and clinical implementation. Moreover, the RGA employs a simplified scoring system, requires no specialized equipment, and is easy to administer, making it particularly suitable for use in nursing homes, community healthcare, and outpatient clinics. Studies have demonstrated that RGA has been widely adopted in Western countries and has exhibited strong psychometric properties in identifying older adults at risk of adverse health outcomes.

The RGA has been translated and validated in Brazil and Spain, where studies have confirmed its strong psychometric properties in identifying older adults at risk of adverse health outcomes [13,14]. However, the RGA has yet been translated or validated for use in China. This study aims to translate and validate the Chinese version of the RGA (C-RGA) while assessing its content validity, construct validity, internal consistency, and test–retest reliability in nursing home residents.

Establishing the psychometric properties of the C-RGA will support its integration into routine geriatric assessments, enhance early screening efficiency, advance personalized health management, and enable targeted interventions in institutional and community healthcare settings. Furthermore, validating this tool will establish a foundation for its potential application in diverse healthcare settings, including outpatient and inpatient care, thereby contributing to the optimization of geriatric health management.

## 2. Materials and Methods

### 2.1. RGA

The Rapid Geriatric Assessment (RGA) is a comprehensive screening instrument designed to assess key geriatric health domains, consisting of four subscales: the simple frail questionnaire screening tool (FRAIL), SARC-F for sarcopenia (SARC-F), the Simplified Nutritional Assessment Questionnaire (SNAQ), and Rapid Cognitive Screen (RCS).

Each subscale independently assesses distinct aspects of geriatric health. The FRAIL scale comprises five questions, with responses scored as 0 (No) or 1 (Yes), where a total score ≥ 3 denotes frailty. It has demonstrated strong predictive validity in detecting frailty among Chinese community-dwelling older adults [15]. The SARC-F comprises five items evaluating strength and mobility, scored as 0 (no difficulty), 1 (some difficulty), or 2 (severe difficulty/assistance required), with a total score ≥4 suggesting probable sarcopenia. Previous validation studies have established that a score of ≥4 predicts sarcopenia and adverse outcomes, including functional decline and falls [16]. The SNAQ assesses appetite and nutritional risk through four questions, with responses scored from one to five, where a total score ≤14 indicates a high risk of ≥5% weight loss within the next six months. Its validity has been substantiated by studies revealing strong correlations with objective markers of malnutrition, such as BMI and serum albumin levels, and its extensive utilization in geriatric settings [17]. The RCS evaluated cognitive function, with scores classified as 8–10 (normal cognition), 6–7 (moderate cognitive impairment), and 0–5 (severe cognitive impairment). As a rapid, straightforward, and sensitive screening tool, the RCS-T has proven effective in bedside consultations and primary care settings for identifying cognitive impairment in older adults, underscoring its utility as an efficient cognitive screening test [18].

Importantly, the RCS serves as a screening instrument rather than a diagnostic tool for dementia; a low RCS score suggests an increased risk of cognitive impairment, but does not establish a clinical diagnosis of dementia. In this study, individuals exhibiting severe cognitive impairment based on RCS were included unless they had a preexisting clinical diagnosis of dementia. This structured framework facilitates a multidimensional assessment of frailty, muscle function impairments, nutritional risk, and cognitive decline, contributing to a comprehensive geriatric evaluation [5].

### 2.2. Study Design

This study adopted a cross-sectional validation design to assess the psychometric properties of the Rapid Geriatric Assessment (RGA) scale, a screening tool used to assess critical domains of geriatric health. The study adhered established best practices in measurement validation, encompassing evaluations of content validity, construct validity, and reliability.

To ensure methodological rigor, we adhered to the consensus-based standards for the selection of health measurement instruments (COSMIN) guidelines, which offer a structured framework for evaluating the measurement properties of health assessment tools. The validation process comprised the following components:

Content Validity: Assessed through an expert panel review, supplemented by quantitative evaluation using the item-level content validity index (I-CVI), the scale-level content validity index (S-CVI), modified kappa (K-modified) [19], and the proportional chance criterion (Pcc).

Construct Validity: exploratory factor analysis (EFA) and confirmatory factor analysis (CFA) were conducted to assess the scale’s dimensionality and model fit.

Reliability: Internal consistency was measured using Cronbach’s α to evaluate the coherence of items within the scale. Split-half reliability was measured using the Spearman–Brown coefficient to assess the consistency between two halves of the scale. Test–retest reliability was analyzed using Pearson’s correlation coefficient over a two-week interval to determine the stability of the RGA scale over time.

### 2.3. Subjects

Participants were enrolled through convenience sampling from a nursing home in Qinghai Province between May and December 2024. This site was chosen due to its large and diverse aging population, as well as the presence of long-term care residents who met the study’s eligibility criteria. The sample size was determined based on Kendall’s Rule, a widely adopted guideline in scale validation research. This approach recommends including 5–10 times the number of questionnaire items to ensure stable psychometric evaluation [20]. To mitigate potential data loss from non-response, an additional 20% sample size adjustment was applied, yielding a final minimum requirement of 228 participants [20]. A total of 416 participants were enrolled, surpassing the recommended threshold. This guaranteed a sufficient sample size for robust exploratory and confirmatory factor analysis (EFA and CFA).

To determine the suitability of the dataset for exploratory factor analysis (EFA) and confirmatory factor analysis (CFA), we employed the Kaiser–Meyer–Olkin (KMO) test and Bartlett’s test of sphericity. A KMO value ≥ 0.6–0.7 and a statistically significant Bartlett’s test of sphericity (*p* < 0.05) verified that the dataset satisfied the necessary assumptions for factor analysis [21,22].

Inclusion criteria: Individuals aged 60 years or older, fully conscious, and capable of providing voluntary informed consent.

Exclusion criteria: Individuals with hearing or speech impairments, clinically diagnosed dementia or mental disorders, or severe illnesses necessitating hospital transfer.

### 2.4. Procedures

#### 2.4.1. Brislin’s Forward and Backward Translation Method

The Rapid Geriatric Assessment (RGA) underwent Brislin’s forward–backward translation method to ensure both linguistic accuracy and cultural equivalence [23]. Two nursing experts, each possessing foreign language proficiency and extensive overseas study experience, independently translated the original scale into Chinese. Any discrepancies between their translations were systematically analyzed and reconciled in the initial Chinese draft.

For the back-translation, an English language expert holding a CATTI certification and a nursing expert with over a decade of overseas experience independently translated the Chinese version back into English without prior access to the original scale. The back-translated version was subsequently cross-examined against the original RGA to detect potential linguistic and conceptual inconsistencies. Any identified discrepancies were thoroughly deliberated upon and iteratively refined by the translation team in close collaboration with geriatrics specialists, ensuring both cultural and clinical relevance. Finally, a native English-speaking medical expert reviewed the back-translated version to verify its fidelity to the original scale.

To enhance the accuracy of cultural adaptation beyond linguistic equivalence, this study followed Beaton’s [19] framework for cross-cultural adaptation, incorporating forward translation, synthesis, back-translation, expert review, and pretesting. The expert panel later assessed the translated version to ascertain semantic clarity, cultural appropriateness, and conceptual alignment.

This rigorous and iterative process resulted in the finalized Chinese version of the RGA (C-RGA), which was subsequently subjected to reliability and validity.

#### 2.4.2. Pre-Survey

Prior to the primary study, a pre-survey was conducted with 30 nursing home residents aged 60 years or older in Qinghai Province. Researchers thoroughly explained the study objectives to participants before administering the questionnaire through face-to-face interactions, following the acquisition of informed consent.

During the pre-survey, researchers observed participants’ responses to assess comprehensibility, documented challenging terms or phrases, and recorded feedback and suggestions. Findings suggested that nursing home residents demonstrated a clear comprehension of the RGA. These findings informed the refinement of the final version of the assessment tool.

#### 2.4.3. Expert Panel

The expert panel consists of six specialists with expertise in gerontology, nursing, psychometric evaluation, and instrument validation. Among them, three experts held doctoral degrees in nursing or public health and had prior experience in developing assessment scales. Two were clinical geriatricians with over 10 years of experience in elderly care, and one was a senior psychometrician specializing in scale adaptation and validation. All panel members had previous experience in the cross-cultural adaptation of health-related measurement instruments, ensuring the methodological rigor of the validation process.

Following Beaton’s cross-cultural adaptation framework, the expert panel conducted an in-depth review of the translated C-RGA to ensure that it was semantically, culturally, and clinically appropriate for the target population. The panel systematically assessed each item based on three key criteria:

Semantic Equivalence: Verifying that the translated wording conveyed the intended meaning of the original text.

Cultural Relevance: Assessing the appropriateness of terminology and phrasing were appropriate within the Chinese socio-cultural context.

Conceptual Alignment: Confirming that the adapted version retained the original intent of each item.

Each item was independently reviewed and rated using a structured 4-point Likert scale (1 = not equivalent, 2 = somewhat equivalent, 3 = mostly equivalent, 4 = fully equivalent). Items a score of ≤2 from any panel member were flagged for revision. Items with discrepancies of ≥1 point among panel members were further discussed in a consensus meeting. Adjustments were made to ensure that the adapted version retained semantic accuracy, cultural appropriateness, and conceptual clarity while aligning with the linguistic and socio-cultural context of the target population.

To further validate the adapted version, the panel evaluated the content validity of each item using the item-level content validity index (I-CVI) and scale-level content validity index (S-CVI). These assessments maintain that the adapted scale maintained its measurement integrity and applicability to the Chinese older adult population.

### 2.5. Statistical Analysis

All statistical analyses were conducted using IBM SPSS Statistics 26.0 and AMOS 24.0. The validity of the scale was assessed through content validity and construct validity: Content validity was assessed using the content validity index (CVI), including item-level CVI (I-CVI) and scale-level CVI (S-CVI) [24]. To strengthen the robustness of content validity assessment, modified kappa (K-modified) [19] and proportional chance criterion (Pcc) were additionally computed to ensure that expert agreement was not attributable solely to chance. Construct validity was investigated using exploratory factor analysis (EFA) and confirmatory factor analysis (CFA). EFA was performed using principal component analysis (PCA) with maximum variance rotation to explore the underlying factor structure of the scale. Sampling adequacy was evaluated using the Kaiser–Meyer–Olkin (KMO) test and Bartlett’s test of sphericity [23]. The model fit in CFA was assessed using fit indices, including the chi-square/degree of freedom ratio ($\chi^{2}/df$), comparative fit index (CFI), the Tucker–Lewis index (TLI), and root mean square error of approximation (RMSEA). Reliability testing was conducted using Cronbach’s alpha coefficient for internal consistency, split-half reliability, and test–retest reliability, ensuring the stability of the four subscales of the C-RGA [14].

### 2.6. Ethics Statement

This study received ethic approval from both the Universiti Kebangsaan Malaysia Ethic Committee and the Ethics Committee of the Nursing Home in Qinghai Province (approval nos: JEP-2024-040 and 20240302).

All procedures complied with the ethical standards of the 1964 Declaration of Helsinki and its later amendments. Participants were fully informed about objectives and significance of the study, including the use of their responses for scientific research. Only participants who provided written informed consent were included in the study.

## 3. Results

Following the standardized translation process, the final Chinese version of the scale was established (Supplementary Materials File S1). The mean completion time for the scale was 3.78 ± 1.46 min, indicating its feasibility and ease of implementation.

Among the 416 participants, 159 (38.2%) were categorized as frail (FRAIL score ≥ 3), indicating a substantial proportion of individuals with functional vulnerability. A total of 188 (45.2%) exhibited probable sarcopenia (SARC-F score ≥ 4), suggesting a high prevalence of muscle dysfunction within this cohort. Additionally, 218 (52.4%) participants were at risk of experiencing over 5% body weight loss in the past six months (SNAQ score ≤ 14), underscoring the burden of malnutrition in institutionalized older adults. In terms of cognitive function, 123 (29.6%) participants demonstrated moderate cognitive impairment (RCS 6–7), whereas 214 (51.4%) exhibited severe cognitive impairment (RCS 0–5), reflecting the high prevalence of cognitive decline within this setting. These findings emphasize the critical need for a multidimensional screening tool capable of systematically evaluating frailty, sarcopenia, nutritional risk, and cognitive impairment among institutionalized older adults.

### 3.1. Demographic Data

A total of 416 participants were included in the study, with a mean age of 73.99 years (SD = 8.11). Further details are presented in Table 1.

### 3.2. Validation Testing

#### 3.2.1. Content Validity

The content validity of the C-RGA scale was assessed through expert consultation, with CVI calculated based on the second round of expert feedback. Six experts evaluated the scale utilizing a four-point Likert scale. For the FRAIL scale, I-CVI for one item was 0.833, whereas all other items attained a perfect score of 1.000. S-CVI/Ave ranged from 0.967 to 1.000 across subscales, with an overall value of 0.982. Expert authority, assessed using judgment criteria and familiarity, yielded an overall authority coefficient of 0.89, signifying robust reliability.

To further verify expert agreement, the modified kappa (K modified) [19,25] and proportional chance criterion (Pcc) were calculated. The results indicated that K modified was 0.881, while Pcc values was 0.722, showing strong inter-rater agreement with minimal likelihood of chance-based concordance.

In addition to assessing content validity, experts recommended several refinements to improve linguistic clarity and cultural relevance. Incorporating their feedback alongside a pilot study involving 30 nursing home residents, the following modifications were implemented:

FRAIL subscale:

The item “Cannot walk one block?” was revised to “the area between two parallel streets (100–300 m)” for better comprehension.

SARC-F subscale:

The item “walking across a room” was revised to “walking from one end of a room to the other” to improve clarity.

The item “climbing a flight of ten stairs” was adjusted to “climbing one story” to align with common Chinese architectural designs, where a typical staircase consists of approximately ten steps per floor.

SNAQ subscale:

The item “I feel full after eating about a third of a meal” was modified to “I feel full before eating half a meal” to better match Chinese expression habits.

RCS subscale:

The diagnosis criterion “dementia” was considered too absolute, so it was changed to “Severe Cognitive Impairment” for greater accuracy.

The item regarding “advance directives” (Do you have an advance medical directive? Yes/No) was removed due to the absence of relevant legal frameworks in China, making the term unfamiliar to nursing home residents.

These refinements, informed by expert consensus and validated through quantitative measures, ensured that the C-RGA scale preserved its conceptual integrity, while remaining culturally adapted for the Chinese older population.

#### 3.2.2. Construct Validity

##### Correlation Analysis of Subscales

Pearson correlation analysis was conducted to explore the interrelationships among Frail, SARC-F, SNAQ, and RCS in older residents. The results demonstrated statistically significant correlations (*p* < 0.01) across all subscales, as summarized in Table 2. FRAIL and SARC-F demonstrated a moderate-to-strong positive correlation (r = 0.582, 95% CI: [0.504, 0.660], *p* < 0.01), suggesting that greater muscle dysfunction is linked to increased frailty. FRAIL and SNAQ exhibited a strong negative correlation (r = −0.671, 95% CI: [−0.742, −0.600], *p* < 0.01), indicating that poor nutritional status is closely associated with frailty progression. FRAIL and RCS showed a moderate negative correlation (r = −0.426, 95% CI: [−0.513, −0.339], *p* < 0.01), implying that cognitive impairment may play a role in frailty development. Similarly, SARC-F and SNAQ displayed a strong negative correlation (r = −0.611, 95% CI: [−0.687, −0.535], *p* < 0.01), reinforcing the association between nutritional deficits and muscle function decline. SARC-F and RCS had a weak-to-moderate negative correlation (r = −0.354, 95% CI: [−0.444, −0.264], *p* < 0.01), suggesting that cognitive decline may exacerbate muscle deterioration. SNAQ and RCS exhibited a moderate positive correlation (r = 0.371, 95% CI: [0.282, 0.460], *p* < 0.01), indicating that better cognitive function is associated with improved nutritional status.

##### Exploratory Factor Analysis (EFA)

The Kaiser–Meyer–Olkin (KMO) test and Bartlett’s test of sphericity were conducted to assess the suitability of the data for factor analysis. The results showed a KMO value of 0.805 and Bartlett’s test statistic of 1825.728, with *df* = 153 and *p* < 0.001. These findings indicate that the data are appropriate for factor analysis.

**Figure 1:**
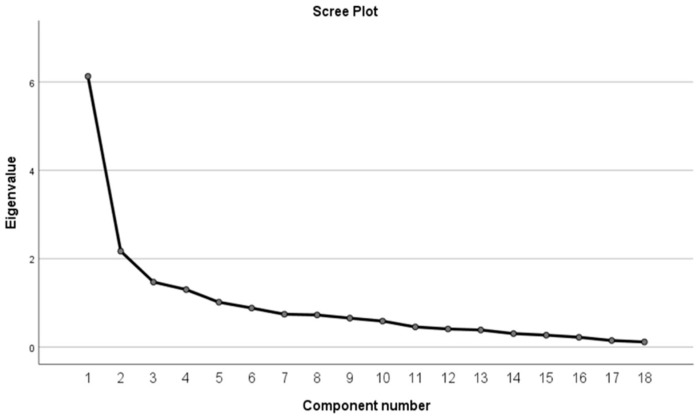
Scree plot for RGA scale. The scree plot was acquired by principal component analysis with correlation matrix. Component number 4 is the elbow point and its eigenvalue is >1. Thus, four factors were extracted.

Exploratory factor analysis (EFA) was conducted via principal component analysis (PCA), employing maximum variance (varimax) rotation to investigate the scale’s factor structure. The scree plot (Figure 1) revealed that when four components were extracted, their eigenvalues were greater than one, with the fourth component representing the inflection point. Accordingly, four factors were retained. Following rotation, the cumulative explained variance reached 61.49%, indicating that these four factors adequately explained the shared variance among the variables [26]. Specifically, factor 1 represents the nutrition dimension (seven items), factor 2 represents sarcopenia (six items), factor 3 represents cognition (three items), and factor 4 represents frailty (two items) (Table 3). Moreover, most items exhibited factor loadings exceeding 0.5 on their corresponding factors, reinforcing the scale’s factorial validity.

##### Confirmatory Factor Analysis (CFA)

CFA was conducted to verify the construct validity of the model. The fit indices obtained from the CFA are presented in Table 4. The CFA results indicated a good model fit, with χ^2^/*df* = 1.122, RMSEA = 0.024, and GFI, AGFI, and CFI all exceeding 0.90. The findings validate the structural integrity of the Chinese version of the RGA, confirming that the original construct of the RGA remains applicable within the Chinese context.

#### 3.2.3. Reliability

The reliability of the C-RGA scale was evaluated through internal consistency, test–retest reliability, and item-level correlation analysis. The overall Cronbach’s α coefficient was 0.839, with subscale values ranging from 0.752 to 0.927, indicating good internal consistency. Test–retest reliability, assessed by re-evaluating 20 randomly selected participants after a 2-week interval, confirmed the external consistency and stability of the scale (Table 5).

## 4. Discussion

### 4.1. Reliability and Validity of the C-RGA

The findings validate that the Chinese version of the Rapid Geriatric Assessment (C-RGA) is a psychometrically robust screening tool for frailty, sarcopenia, nutritional risk, and cognitive function among nursing home residents. Given that the RGA comprises four independently scored, well-established subscales (SNAQ, SARC-F, FRAIL, and RCS), its overall internal consistency cannot be directly evaluated. Therefore, reliability evaluation was conducted separately for each subscale. Nonetheless, its high test–retest reliability (>0.800) confirms its temporal stability, rendering it suitable for both clinical and research applications.

A major strength of the C-RGA is its structural validity, as demonstrated by confirmatory factor analysis (CFA), which validated the four-domain model ($\chi^{2}/df$ = 1.122, RMSEA = 0.024, with GFI, AGFI, and CFI all exceeding 0.90). These findings support the conceptualization of SNAQ, SARC-F, FRAIL, and RCS as distinct but interrelated components, reflecting key geriatric health concerns. The high content validity index (CVI: I-CVI > 0.80, S-CVI/Ave = 0.982) further validates that the translated items effectively capture the intended constructs.

Compared to previous studies, the C-RGA subscales demonstrated strong reliability [15,27,28,29,30], particularly the SNAQ subscale (Cronbach’s α = 0.927), which demonstrated superior internal consistency relative to earlier versions [5,10,30]. This highlights the robustness of SNAQ in detecting nutritional risk, a critical determinant in sarcopenia and frailty progression. Despite its traditionally lower reliability, the FRAIL subscale remains clinically valuable due to its ability to rapidly screen for frailty-related risks in institutionalized older adults. Similarly, the Chinese version of the Japan Frailty Scale (JFS-C) has also been validated for frailty screening, demonstrating good reliability and validity in identifying frailty risk [12].

While both C-RGA and JFS-C assess frailty, the C-RGA expands upon this by incorporating nutritional risk, sarcopenia, and cognitive function, allowing for a more comprehensive evaluation of geriatric health. Compared to traditional frailty screening tools, such as JFS-C [12], the C-RGA provides a multidimensional framework that may enhance early risk identification and intervention planning. Additionally, more extensive geriatric assessment tools, such as the Comprehensive Geriatric Assessment (CGA), offer a thorough evaluation of older adults’ health but may not be feasible for large-scale screening due to their complexity and time requirements. The C-RGA provides a structured and multidimensional screening tool that offers both efficiency and comprehensive geriatric assessment, making it particularly suitable for large-scale screening in institutional and primary care settings. Future studies should further compare the predictive validity and clinical applicability of these tools in diverse aging populations.

### 4.2. Factor Structure and Implications

The EFA results and expert consensus indicated that the specific assignment of certain items to subscales was modified, without altering the original item content or overall structure of the scale. Despite the reallocation of certain items across factors, the overall structure remained consistent with the original RGA model.

To improve the sensitivity of the nutritional risk (SNAQ) subscale in detecting malnutrition-related frailty and functional decline, three items were reassigned. The item assessing unintentional weight loss (“Have you lost more than 5% of your weight in the last six months?”) was transferred from the FRAIL subscale to SNAQ, as weight loss is a key indicator of nutritional deterioration and increased frailty risk [31,32,33]. Furthermore, “Do you feel fatigued?”, originally part of FRAIL, was reassigned to SNAQ, given that inadequate energy intake is a major cause of fatigue and physical decline [3]. Lastly, “Remember five items and recall them later?”, previously categorized under RCS, was moved to SNAQ to reflect the strong association between cognitive function and dietary behavior regulation [34]. Studies suggest that deficits in memory and executive function contribute to appetite loss and malnutrition, thereby supporting this reassignment [35].

To improve the sarcopenia (SARC-F) subscale’s ability to assess muscle function impairments, two items were reassigned. The item assessing mobility decline (“Are you unable to walk 200–300 m?”) was transferred from FRAIL to SARC-F, recognizing that impaired walking ability is a primary symptom of sarcopenia and reflects muscle weakness. Additionally, the item “Do you currently have more than five chronic diseases?” was moved from FRAIL to SARC-F, as chronic multimorbidity is strongly linked to muscle atrophy, reduced strength, and sarcopenia progression. These adjustments ensure that SARC-F better captures mobility-related functional decline associated with sarcopenia [2].

Following these refinements, the finalized structure of the C-RGA consists of seven items in SNAQ, six in SARC-F, three in RCS, and two in FRAIL. These modifications reinforce the scale’s ability to detect malnutrition-related frailty, sarcopenia progression, and functional decline in older adults. Given the strong correlation between nutritional risk (SNAQ) and sarcopenia (SARC-F) (*p* < 0.01), these refinements further enhance the scale’s utility in detecting at-risk individuals and guiding early interventions.

### 4.3. Association Between Malnutrition, Frailty, Sarcopenia, and Cognitive Impairment

The observed correlations among the four C-RGA subscales reinforce its clinical relevance, aligning with previous findings that frailty, sarcopenia, nutritional risk, and cognitive impairment frequently co-exist in older adults [13,14]. The strong negative correlation between SNAQ and both FRAIL (r = −0.671, *p* < 0.01) and SARC-F (r = −0.611, *p* < 0.01) suggests that nutritional deficits are closely associated with increased frailty and muscle dysfunction. However, given the moderate effect sizes, these findings indicate that while poor nutrition is a significant risk factor, frailty and sarcopenia likely result from a combination of physiological, behavioral, and environmental influences. This underscores the importance of incorporating routine nutritional screening into geriatric assessments to facilitate early intervention and mitigate functional decline [36,37,38].

Furthermore, the strong positive correlation between FRAIL and SARC-F (r = 0.582, *p* < 0.01) suggests a substantial overlap in their underlying physiological mechanisms, particularly in muscle deterioration and functional decline. While this correlation is statistically strong, it remains a single contributing factor, as frailty is influenced by a broader range of physiological, metabolic, and behavioral factors beyond sarcopenia alone. These findings reinforce the necessity of integrating frailty and sarcopenia assessments in geriatric screenings, as relying solely on muscle function assessments may not comprehensively capture an individual’s frailty risk. Therefore, a multidimensional evaluation incorporating nutritional and cognitive assessments remains essential to identifying older adults at the highest risk for functional impairment and guiding targeted interventions [39].

Notably, cognitive function (RCS) exhibited a significant negative correlation with frailty (r = −0.426, *p* < 0.01) and sarcopenia (r = −0.354, *p* < 0.01), while displaying a positive correlation with nutritional status (r = 0.371, *p* < 0.01). These findings suggest that cognitive impairment may be associated with frailty and sarcopenia progression, but the observed moderate effect sizes indicate that its clinical relevance may be limited. This association may be influenced by additional factors such as systemic inflammation, socioeconomic factors, and chronic disease burden, highlighting the need for a comprehensive assessment approach. Given the moderate effect sizes, its clinical relevance may be limited in predicting frailty and sarcopenia progression. While the associations are statistically significant, the moderate effect sizes suggest that cognitive impairment alone may not be a strong predictor of frailty or sarcopenia, highlighting the need for a multifactorial risk assessment approach. Cognitive impairment is likely one of multiple contributing factors rather than a primary determinant, and its predictive value may vary depending on an individual’s overall health status and comorbidities [40]. These results reinforce the importance of integrating cognitive and nutritional assessments in routine geriatric screenings to identify individuals at heightened risk of functional decline and implement early interventions aimed at mitigating frailty and sarcopenia progression.

Given the observed associations, the C-RGA’s ability to concurrently assess frailty, sarcopenia, nutritional risk, and cognitive function underscores its potential clinical utility as a comprehensive geriatric screening tool. While these findings indicate that C-RGA has the potential to facilitate the early identification of at-risk individuals and support timely interventions, its clinical utility should be interpreted alongside its moderate effect sizes. Nevertheless, given its ability to concurrently assess frailty, sarcopenia, nutritional risk, and cognitive function, C-RGA remains a promising screening tool for use in geriatric assessments. Further validation in larger and more diverse populations is warranted to establish its broader applicability.

Future studies should investigate whether integrating additional biomarkers or longitudinal assessments could enhance its predictive accuracy and practical applicability. As this study was conducted in a single nursing home with a cross-sectional design, the generalizability of these findings to other populations remains uncertain. Future research should investigate whether the observed relationships hold in community-dwelling older adults and other institutional settings, and whether the effect sizes observed in this study translate into meaningful clinical outcomes. Additionally, longitudinal studies are needed to evaluate the sustained impact of early nutritional interventions on mitigating frailty and sarcopenia progression, particularly among institutionalized older adults.

### 4.4. Practical Applications of C-RGA in Nursing Homes

The findings of this study support the practical relevance of the C-RGA as a rapid screening tool for frailty, sarcopenia, nutritional risk, and cognitive function in nursing home residents. Given the high prevalence of malnutrition-related frailty and sarcopenia in institutionalized older adults, the strong correlations observed between nutritional status (SNAQ), muscle function decline (SARC-F), and frailty progression (FRAIL) highlight the potential clinical value of a multidimensional geriatric screening tool in long-term care settings.

While this study focused on the translation and validation of the C-RGA, the results suggest that integrating this tool into routine assessments could facilitate the early identification of at-risk individuals and support targeted intervention strategies. These implications are consistent with existing international efforts to adapt the RGA for use in different healthcare contexts. For example, a Spanish version of the RGA has been translated and validated for use in long-term care settings in Mexico (Use of the Spanish Version of the Rapid Geriatric Assessment in Mexican Patients in Long-Term Care). This adaptation underscores the global applicability of the RGA in various healthcare environments. The successful implementation of the Spanish RGA in Mexican long-term care institutions further demonstrates the feasibility of integrating culturally adapted versions of the tool for early frailty and sarcopenia detection in different linguistic and healthcare contexts [13]. Moreover, Brazilian researchers have validated the Brazilian version of the RGA, demonstrating its strong reliability and validity, thereby confirming its applicability in geriatric assessments within the Brazilian healthcare context [14]. The C-RGA follows this trend, offering a validated Chinese version specifically designed for use in Chinese nursing home residents.

Future research should focus on assessing the predictive validity of the C-RGA in tracking frailty progression, nutritional status deterioration, and sarcopenia-related muscle function decline over time. Considering the robust correlations observed between nutritional risk (SNAQ), sarcopenia (SARC-F), and frailty (FRAIL), longitudinal studies are needed to determine whether early nutritional risk assessment can predict worsening physical function and increased frailty severity. Additionally, further validation of the C-RGA across diverse healthcare settings—including community-dwelling older adults and hospitalized patients—would be instrumental in confirming its broader applicability for early geriatric risk screening and targeted interventions.

### 4.5. Strengths and Limitations of the Study

This study represents the first systematic translation and validation of the Rapid Geriatric Assessment (RGA) in a sample of institutionalized older adults in China, confirming its strong psychometric properties within this study population. The C-RGA demonstrated high internal consistency, supporting its robustness as a screening tool for frailty, sarcopenia, nutritional risk, and cognitive function. Notably, the SNAQ subscale exhibited the highest internal consistency, reinforcing its reliability in detecting nutritional risk and its potential role in early malnutrition intervention strategies. The strong correlation between SNAQ and SARC-F further supports the integration of nutritional assessment in frailty and muscle function screening, though future validation is required in a broader population.

However, several limitations should be noted. This study was conducted in a single nursing home in Qinghai, a region with relatively limited healthcare resources and elderly care facilities, which may affect the generalizability of the findings to other institutional settings and urban healthcare systems. Future studies should expand the sample size through multicenter validation in regions with diverse healthcare infrastructures, including community-based primary care, hospitals, and long-term care facilities, to assess the tool’s adaptability across different populations. Additionally, the cross-sectional design does not allow for the evaluation of the C-RGA’s predictive validity over time. Longitudinal studies are needed to determine its ability to track frailty progression, sarcopenia development, and changes in nutritional and cognitive status.

## 5. Conclusions

This study successfully translated and validated the Rapid Geriatric Assessment (RGA) scale, demonstrating that C-RGA has a well-structured framework, high reliability, and strong validity, supporting its potential use as a reliable, effective, and convenient tool for comprehensive geriatric assessment. However, due to regional and sample size limitations, further validation is required in various healthcare settings, including community-based primary care, hospitals, and long-term care facilities, to determine the broader applicability of the C-RGA. Additionally, longitudinal studies are needed to evaluate its predictive accuracy in tracking frailty progression, sarcopenia development, and changes in nutritional and cognitive status over time.

## Figures and Tables

**Table 1:** Descriptive statistic of nursing home residents.

Item	Category	*n*	Proportion (%)
Age (years)	60–69	120	28.8
	70–79	170	40.9
	≥80	126	30.3
Gender	Male	327	78.6
	Female	89	21.4
Education level	Primary school and below	290	69.7
	Junior high school/technical secondary	87	20.9
	Senior high school/junior college	30	7.3
	Undergraduate or above	9	2.1
Ethnicity	Han	392	94.2
	Minority	24	5.8
Marital status	Married	260	62.5
	Widowed	110	26.4
	Divorced	30	7.2
	Never married	16	3.8
Occupation	Government/institution	100	24.0
	Professional/technical	50	12.0
	Clerk/service	60	14.4
	Agriculture	40	9.6
	Manufacturing/transportation	50	12.0
	Military	10	2.4
	Others	26	6.2
	Unemployed	80	19.2
Source of income	Pension	250	60.1
	Family support	120	28.8
	Low-income assistance	30	7.2
Medical insurance type	Urban employee basic medical insurance	150	36.1
	Urban and rural resident medical insurance	200	48.1
	Social assistance medical insurance	40	9.6
	Self-funded medical	26	6.2

**Table 2:** Pearson correlation analysis among FRAIL, SARC-F, SNAQ, and RCS.

	FRAIL	SARC-F	SNAQ	RCS
FRAIL	1.000			
SARC-F	0.582 **	1.000		
SNAQ	−0.671 **	−0.611 **	1.000	
RCS	−0.426 **	−0.354 **	0.371 **	1.000

** *p* < 0.01.

**Table 3:** C-RGA factor analysis results after rotation—component matrix.

Item	Component			
	1	2	3	4
Are you fatigued?	0.693	−0.028	0.014	−0.151
Food taste	0.693	−0.216	−0.089	0.340
My appetite is	0.691	−0.258	0.006	0.314
Cannot walk one block? (100–300 m)	0.662	−0.266	−0.050	−0.448
When I eat (e.g., feeling full after just a few bites)	0.642	−0.438	−0.074	0.288
How much difficulty do you have climbing one story?	0.638	−0.363	0.014	0.168
Remember and memory these five objects	0.602	−0.113	0.270	0.219
Normally I eat	0.399	0.537	−0.110	0.356
How many times have you fallen in the last year?	−0.378	0.620	0.276	−0.016
How much difficulty do you have walking from one end of a room to the other?	−0.093	0.867	−0.158	−0.075
How much difficulty do you have transferring from a chair or bed?	−0.168	0.838	−0.142	−0.087
How much difficulty do you have climbing one story?	−0.324	0.741	0.091	−0.085
How much difficulty do you have in lifting and carrying 10 pounds (4.5 kg)?	−0.375	0.641	0.059	−0.028
Clock draw * (clock face)	0.032	−0.048	0.877	−0.015
Clock draw * (time marking)	0.116	−0.181	0.834	0.017
Tell a story and answer the question	0.466	−0.164	0.576	0.197
Have you lost more than 5% of your weight in the last 6 months?	0.136	0.054	−0.096	0.791
Do you have more than five illnesses?	0.092	−0.345	0.059	0.657
Initial eigenvalues	6.127	2.170	1.471	1.301
Cumulative explained variance % after rotation	21.685	39.913	51.267	61.497
Rotated eigenvalues	3.903	3.281	2.044	1.841

* Clock draw: Provide older residents with a blank sheet of paper featuring an empty clock face and ask them to draw the clock’s numbers and place the hands to indicate 10:45.

**Table 4:** Construct validity.

	χ^2^/*df*	RMSEA	GFI	AGFI	CFI	TLI
Ideal	≤3.0	≤0.05	≥0.90	≥0.90	≥0.90	≥0.90
Acceptable	≤5.0	≤0.08	≥0.80	≥0.80	≥0.80	≥0.80
Results	1.122	0.024	0.929	0.906	0.905	0.888

**Table 5:** Reliability of the C-RGA subscales.

Subscales	Cronbach’s α	Split-Half Reliability	Test–Retest Reliability
FRAIL	0.752	0.726	0.850
SARC-F	0.874	0.852	0.892
SNAQ	0.927	0.913	0.938
RCS	0.823	0.806	0.826

## Data Availability

The data presented in this study are available on request from the corresponding author. The data are not publicly available due to privacy restrictions.

## Supplementary Materials

The following supporting information can be downloaded at: https://www.mdpi.com/article/10.3390/nu17050873/s1, File S1: The Chinese version of RGA (C-RGA).
