# Supplementary material for: Translation and Validation of the Chinese Version of the Rapid Geriatric Assessment (C-RGA): A Screening Tool for Geriatric Syndromes in Nursing Home Residents
Source: Nutrients. 2025 Feb 28;17(5):873. doi: 10.3390/nu17050873 (PMC11901678; doi:10.3390/nu17050873)
Supplement: Supplementary file 1 [file nutrients-17-00873-s001.zip › nutrients-3491730-supplementary.pdf]

Saint Louis University
Chinese version of the Rapid Geriatric Assessment (C-RGA)

The simple "FRAIL" questionnaire Screening tool (FRAIL)

Fatigue: Are you fatigued?

Resistance: Cannot walk up one flight of stairs?

Aerobic: Cannot walk the area between two parallel streets (100–300 m)?

Illnesses: Do you have more than 5 illnesses?

Loss of weight: Have you lost more than 5% of your weight in the last 6 months?

3 or greater = frailty; 1 or 2 = prefrailty

Scorie：___________

From Morley JE, Vellas B, Abellan van Kan G, et al. J Am Med Dir Assoc 2013; 14:392-397

SARC-F for sarcopenia (SARC-F)

**S**trength	How much difficulty do you have in lifting and carrying 10 pounds (4.5kg)?

Scoring: None = 0; Some = 1; A lot or unable = 2

**A**ssistance in walking	How much difficulty do you have walking from one end of a room to the other?

None = 0; Some = 1; A lot, use aids or unable = 2

**R**ise from a chair	How much difficulty do you have transferring from a chair or bed?

None = 0; Some = 1; A lot or unable without help = 2

**C**limb stairs	How much difficulty do you have climbing one story?

None = 0; Some = 1; A lot or unable = 2

**F**alls	How many times have you fallen in the last year?

None = 0; 1-3 falls = 1; 4 or more falls = 2

Total score of 4 or more indicators Sarcopenia

Scorie：___________

From From Malmstrom TK, Morley JE, J Frailty and Aging 2013: 2:55-6.

Simplified Nutritional Assessment Questionnaire (SNAQ)

**My appetite is**	**When I eat**
a.Very poor	a. I feel full after eating only a few mouthfuls
b. Poor	b.I feel full before eating half a meal
c. Average	c. I feel full after eating over half a meal
d. Good	d. I feel full after eating most of the meal
e. Very good	e. I hardly ever feel full
**Food tastes**	**Normally I eat**
a.Very bad	a. Less than one meal a day
b. Bad	b. One meal a day
c. Average	c. Two meals a day
d. Good	d. Three meals a day
e. Very good	e. More than three meals a day

Scoring: a=1; b=2; c=3; d= 4, e=5

A score≤14 indicates significant risk of at least 5% weight loss within 6 months

Scorie：___________

From Wilson et al. Am J Clin Nutr 2005;82:1074-81

Rapid Cognitive Screen (RCS)

1.Please remember these five objects. I will ask you what they are later.

[Read each object to patient using approx.. 1 second intervals]

Apple Pen Tie House Car

2.[Give patient pencil and the blank sheet with clock face.] This is a clock face

Please put in the hour markers and the time at ten minutes to eleven o'clock. [2 pts/hr markers ok; 2 pts/time correct]

3.What were the five objects I ask you to remember? [1 pt/ea]

4.I'm going to tell you a story. Please listen carefully because afterwards, I'm going to ask you about it.

Alan was a very successful stockbroker. Later, she met Ah San, an extremely handsome man. They married and had three children. They lived in Xining. Alan later quit her job and stayed at home to take care of the children. When the children became teenagers, she returned to the workplace. From then on, they lived happily together.

What province did they live in? [1 pt]

8-10: Normal; 6-7:Mild Cognitive lmpairment;0-5: Severe cognitive impairment

Scorie：___________

From Malmstrom Tk, Voss VB, Cruz-Oliver DM etal I Nutr HealthAging 2015:19:741-744
